# The Microbiota-Bone-Allergy Interplay

**DOI:** 10.3390/ijerph19010282

**Published:** 2021-12-28

**Authors:** Maria Maddalena Sirufo, Francesca De Pietro, Alessandra Catalogna, Lia Ginaldi, Massimo De Martinis

**Affiliations:** 1 Department of Life, Health and Environmental Sciences, University of L’Aquila, Piazzale Salvatore Tommasi n. 1, 67100 L’Aquila, Italy; maddalena.sirufo@gmail.com (M.M.S.); fra722@hotmail.it (F.D.P.); alessandra.cat4@gmail.com (A.C.); lia.ginaldi@cc.univaq.it (L.G.); 2 Allergy and Clinical Immunology Unit, Center for the Diagnosis and Treatment of Osteoporosis, AUSL 04, 64100 Teramo, Italy

**Keywords:** microbiota, gut microbiota, allergy, osteoporosis, bone metabolism, food allergy, skeletal health, inflammation, osteoimmunology

## Abstract

Emerging knowledge suggests an increasing importance of gut microbiota in health and disease. Allergy and bone metabolism are closely interconnected, and the possible negative effects of common therapies are not the only aspects of this relationship. The immune system is influenced by the microbiota-host interactions, and several pieces of evidence suggest the existence of an interplay between microbiota, bone metabolism, and allergies. Understanding these inter-relationships is essential for the development of new potential strategies of treatment and prevention targeting microbiota. A wide range of substances and germs, prebiotics and probiotics, are capable of influencing and modifying the microbiota. Prebiotics and probiotics have been shown in several studies to have different actions based on various factors such as sex, hormonal status, and age. In this review, we summarize the latest knowledge on the topic, and we discuss practical implications and the need for further studies.

## 1. Introduction

**Figure 1:**
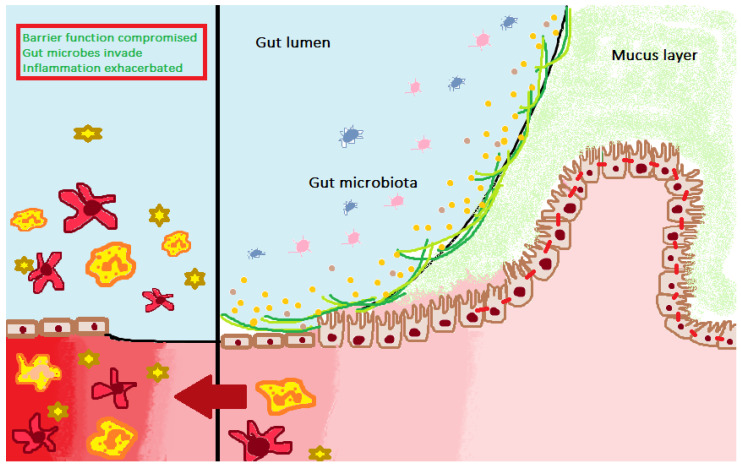
The human gastrointestinal tract is colonized by about 10^13^–10^14^ microorganisms, of which 15,000 different bacterial strains are located mainly in the colon in a symbiotic relationship with the host. In normal conditions, the microbiota is characterized by the predominance of obligate anaerobic members of Firmicutes and Bacteriodetes phyla, which guarantee intestinal and general health, while the loss of homeostasis, dysbiosis, linked to the proliferation of some bacterial populations such as the Enterobacteriaceae or the absence of important commensal bacteria helps to create a more favorable environment for the growth of pathogens, predisposing the organism to pathological conditions.

The term microbiota defines the whole of microorganisms, not only bacteria but also fungi, protozoa, and viruses present in our organism. In particular, the human gastrointestinal tract is colonized by about 10^13^–10^14^ microorganisms, of which 15,000 different bacterial strains are located mainly in the colon in a symbiotic relationship with the host [1]. In normal conditions, the microbiota is characterized by the predominance of obligate anaerobic members of *Firmicutes* and *Bacteroidetes* *phyla*, which guarantee intestinal and general health, while the loss of homeostasis, known as dysbiosis, is linked to the proliferation of some bacterial populations such as the Enterobacteriaceae or the absence of important commensal bacteria helps to create a more favorable environment for the growth of pathogens, predisposing the organism to pathological conditions [2,3] (Figure 1). According to what was recently reported in a study by the Human Microbiome Project and the European consortium Meta HIT, the human intestinal bacterial flora, despite being composed of a very high number of different species, can be divided into three most represented genus: *Bacteroides* and *Prevotella*, belonging to the phylum *Bacteroidetes,* and *Ruminococcus* belonging to the phylum *Firmicutes* [4]. In the time from birth to adulthood, the microbiota undergoes numerous changes; in fact, the neonatal microbiota is precociously formed by *Escherichia coli* of the birth canal followed by *Bacteroides, Bifidobacteria*, and *Clostridium* in the first week of life and reaches stabilization already appearing similar to that of adults only around 2–3 years old [5]. The intestinal microbiota has many important functions for maintaining the health of the host, such as the formation and maintenance of the intestinal barrier, through the production of short-chain fatty acids (SCFAs) resulting from the fermentation of undigested nutrients, immunostimulation, and immunotolerance, synthesis of substances, metabolic-trophic function, metabolism of drugs and toxins [6]. The balance between the intestinal microbiota and the host is maintained through various mechanisms, including the secretion of gastric acid, mucus, bile salts, and mucous Ig, mucosal pH, intestinal motility, local and systemic immunity, and interactions between different microbial species. An alteration in the microbiota-host relationship could potentially cause the onset of gastrointestinal or extra-intestinal diseases, defined as “intestinal microbiota related diseases”. Among the most known, we remember allergic diseases, inflammatory bowel diseases, obesity, metabolic syndrome, type 1 and 2 diabetes, cardiovascular disease and even osteoporosis (OP) [1].

## 2. Osteoporosis

OP is defined as a systemic and metabolic bone disease characterized by a decreased bone mass per unit of volume and a deterioration of the microstructure of the bone tissue, thus increasing the risk of fracture caused by bone fragility. OP is a female-dominated disease more common in postmenopausal women, with a male/female ratio of 1:6 [7,8]. In physiological conditions, there is a balance between osteogenesis, promoted by osteoblasts, and bone resorption by osteoclasts, regulated by a complex molecular mechanism in which estrogens, parathyroid hormone, vitamin D, and inflammatory cytokines are important factors [9,10,11,12]. Osteoblasts secrete the nuclear factor receptor activator ligand κB (RANKL), which interacts with the RANK receptor, a member of the tumor necrosis factor family expressed by osteoclasts and their precursors. The RANK/RANKL interaction, which promotes the differentiation and survival of osteoclasts, is controlled by the soluble decoy receptor Osteoprotegerin (OPG), a natural inhibitor of RANKL. The alteration of these mechanisms, therefore, leads to the prevalence of the RANK /RANKL interaction and to increased bone resorption with consequent OP [13,14,15,16]. It has been observed that the loss of estrogens, a condition typical of the postmenopausal state, increases the expression of proinflammatory cytokines, namely interleukin (IL)-1, IL-6, IL-7, IL-17 TNFα, Macrophage colony-stimulating factor (MCSF), and RANKL by osteoblasts, T cells and B cells. Among these cells, T helper lymphocytes (Th)17 are thought to play a particularly critical role in bone loss associated with estrogen deficiency while, regulatory T cells (Tregs) are capable of producing different cytokines, including Transforming Growth Factor (TGF) beta 1, IL-4 and IL-10, inhibiting bone resorption, and reducing the production of effector cytokines [17,18,19,20,21,22].

## 3. Microbiota and Osteoporosis

The microbiota can, through the regulation of mineral absorption of calcium, phosphorous, and magnesium and the production of incretins, serotonin, and gut-derived factors, influence the health of bone. Many studies have already shown that the intestinal microbiome is closely related to bone metabolism, the absorption of bone minerals in physiological conditions, and to the pathogenesis of OP [8,23,24,25].

In the studies carried out by Collins et al., it was observed that bone mass was higher in germ-free (GF) mice than in conventional mice; GF mice also had a reduced number of osteoclasts per bone surface and a reduced frequency of CD4+ T cells and osteoclast precursors in their bone marrow [23]. These results varied following colonization of the germ-free gut with a conventional microbiota, suggesting the beneficial action of probiotics in the prevention of OP [26].

Uchida et al. found that, comparing the primary osteoblasts isolated from alveolar bones and scalps of the GF mice and the osteoblasts isolated from the specific pathogen-free (SPF) mice, the last expressed substantially more osteocalcin, alkaline phosphatase (ALP), and insulin-like growth factor-I/II (IGF-I/IGF-II), with a decreased ratio of OPG/RANKL. In the end, the bone density of SPF mice was lower than GF mice, indicating that the gut microbiome has a greater regulatory impact on osteoclasts and bone density [27].

Another important study conducted by Jing-Jing Ni et al. about the valuation of the causal relationship between gut microbiota to bone mineral density (BMD) discovered that an increase in the *Clostridiales* class and in the *Lachnospiraceae* family was negatively correlated to BMD, demonstrating the causal relationship between microbiota and bone development [28]. 

Significant changes in the intestinal microbiota were observed in patients with OP. In fact, while in healthy controls, the composition of the intestinal microbiota was given by the maximum presence of *Bacteroides*, *Faecalibacterium*, and *Prevotella*, in OP patients, it was possible to observe a variation of the bacterial composition with an increase in the proportion of *Firmicutes* and a decrease in the proportion of *Bacteroidetes* than in healthy people [29].

The diet is therefore essential for the absorption of nutrients and for the composition of the microbiota; high consumption of fats is associated with a reduction in the Bacteriodetes/Firmicutes ratio and metabolic imbalances for the host, as found in patients with OP. On the contrary, a low-calorie diet removes harmful substances, leading to beneficial effects for the host [30,31]. An important role concerning the composition of the intestinal microbiota is played, also, by the use of antibiotics. Prolonged therapy can change the normal composition of the bacterial flora, altering its biological metabolism. This leads to impaired intestinal absorption, especially a deficiency of minerals important for bone health, thus contributing to the development of OP [32].

The link between the microbiota and BMD is now established; in particular, bacterial overgrowth has been associated with malabsorption and the consequent alteration in the metabolism of calcium, carbohydrates, vitamins B and K, essential elements for bone metabolism. Furthermore, a high concentration of probiotics *Lactobacillus reuteri* and *Bifidobacterium longum* facilitate the absorption of calcium, magnesium, and phosphorus, increasing BMD. Some species of Lactobacilli, intervening in the degradation of proteins present in milk, are the main ones responsible for the beneficial effects of milk found in bone health [33,34,35].

Alterations of the microbiota are able to lead to a dysfunction of the intestinal barrier with an increase in serum lipopolysaccharide (LPS) and consequent increase in intestinal permeability and osteoclasts survival [36,37].

Moreover, the intestinal microbiota is able to influence bone metabolism both directly, through the production of SCFAs, above all butyrate, and through the influence on metabolic hormones such as serotonin, an important factor in the development and maintenance of bone.

**Figure 2:**
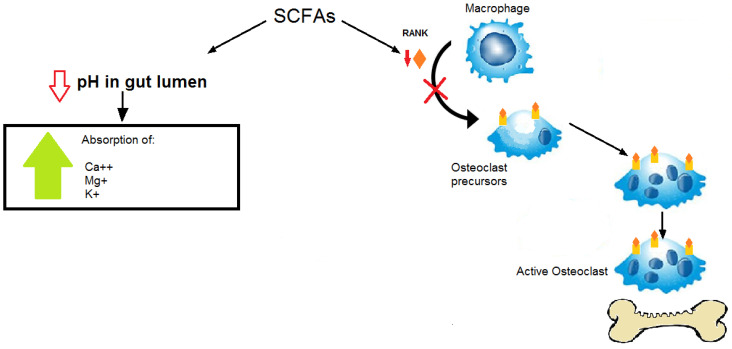
SCFAs play a very important role in bone formation and mineralization by acting on the osteoprotegerin pathway, reducing osteoclastogenesis by suppressing the RANKL pathway, and reducing the pH of the intestinal tract by subsequently increasing the absorption of minerals.

SCFAs play a very important role in bone formation and mineralization, acting on the osteoprotegerin pathway, suppressing the RANKL pathway, and influencing the glucagon-like peptide 1, involved in osteoblast-adipocyte differentiation of bone mesenchymal stem cells [38,39,40,41,42,43] (Figure 2).

A mechanism underlying the changes in the gut microbiota in patients with OP has been hypothesized to involve the immune-inflammatory axis as a key bridge linking the intestinal microbiota to bone metabolism [44,45,46,47,48]. 

In particular, the microbiota can increase TNF-α, one of the activators of the RANK-RANKL pathway, which leads to increased bone resorption by altering bone homeostasis in mice [23]. 

Finally, the microbiota also appears to influence flavonoids and diethylstilbestrol, estrogens of intestinal origin, whose alteration influences bone homeostasis, being the estrogen deficiency directly involved in the risk of postmenopausal OP [49,50].

It is possible to hypothesize the modulation of the microbiota as a therapy limiting the progress of the OP in addition to conventional therapies. One of the strategies that can be used is the administration of probiotics, live microorganisms that restore intestinal permeability, improve the immune barrier function of the intestine, promote the production of IgA, and inhibit the release of proinflammatory cytokines. Several studies were conducted to evaluate the beneficial action of probiotics on the prevention of primary OP, highlighting complex bone protection mechanisms.

Particularly in vitro studies, *Lactobacillus reuteri* was able to inhibit the differentiation of osteoclasts from monocytic macrophages, releasing an anti-osteoclastogenic factor capable of modulating osteoclastogenesis [51]. Furthermore, a secreted component of *Lactobacillus reuteri* was sufficient to inhibit TNFα-induced suppression on pre-osteoblastic cells [52]. It has also been shown that *Lactobacillus reuteri* secretes histamine, capable of suppressing the production of TNFα by human monocytoid cells [53]. *Lactobacillus helveticus* and *Lactobacillus casei* have a direct effect on bone cells [54]. In particular, the addition of *Lactobacillus helveticus* fermented milk products to primary bone marrow cultures showed an increased calcium accumulation in osteoblast cultures, suggesting its role as an enhancer in osteoblast differentiation [55]. Both *Lactobacillus rhamnosus GG* (LGG) and the commercially available probiotic supplement reduce expression of TNFα, IL-17, and RANKL in cells isolated from the small intestine and bone marrow in mice who underwent ovariectomy [56].

The mechanisms by which probiotics act in human cells are very complex and not fully explored. The direct action of probiotics on osteoclasts must be considered limited in humans, while probably a key role is played in the intestine. Bacteria have been shown to be essential for the synthesis of numerous vitamins and enzymes required for matrix formation and bone growth, including Vitamin D, K, C, and folate. Furthermore, bacteria of the genus *Bifidobacteria* are capable of producing SCFA, which can reduce the pH of the intestinal tract by subsequently increasing the absorption of minerals. Some studies demonstrate that *Lactobacillus reuteri* is able to suppress the gene expression of proinflammatory and pro-osteoclastogenic cytokines, both in the intestine and in the bone marrow. Probiotic bacteria can directly increase calcium transport across the intestinal barrier by reducing intestinal inflammation [25,51,57,58,59,60,61]. 

The results showed that probiotic preparations prevent increased intestinal permeability caused by the depletion of sex steroids, thus limiting the production of osteoclasts. This serves as a proof of concept that the gut microbiome and probiotic preparations are involved in trabecular bone loss caused by sex steroid deficiency [8]. 

## 4. Allergy

Food allergy (FA) is an unexpected reaction resulting from an immunological alteration, in which a specific and reproducible immune response is triggered by the ingestion of food antigens normally tolerated by the population [62]. 

Although the prevalence rate of FA is variable in relation to age and geographical location, about 10% of the population is affected by FA, with a prevalence in childhood and in developing countries [63,64,65]. All foods can cause FA, but the most commonly involved are peanuts, cow’s milk, hen’s egg, tree nuts, fish, shellfish, wheat, seeds, and soy [66]. The essential process for avoiding the development of FA is oral tolerance. It derives from oral exposure to food antigens and is mediated by dendritic cells (DCs), able to stimulate the differentiation of naive T cells into positive forkhead box P3 (Foxp3) T cells that produce IL-10, leading to the inhibition of sensitization to specific food allergens. Proinflammatory cytokines produced by intestinal epithelial cells in association with pathogen-associated molecular patterns (PAMPs) or damage-associated molecular patterns (DAMPs) lead to the production of inflammatory cytokines that switch antigen-presenting cells into a proinflammatory phenotype, increasing Th2 cells, that drive the allergic response through the production of IL-4, the expansion of eosinophils and mast cells and the isotypic switch of B cells towards the production of IgE. The onset of FA is linked to the breakdown of oral tolerance. The first phase of sensitization, at the first contact with the antigen, leads to the production of specific IgEs, which are anchored to specific receptors on mast cells and basophils. At the second contact with the allergen, the cells are activated by the binding of the antigen to the IgEs, releasing various mediators including histamine, TNF-α, platelet activation factor (PAF), leukotrienes, IL-4, IL-5, IL-9, IL-13, IL-31, and IL-33, which lead to a range of symptoms from the skin to life-threatening one [67,68].

Increasing evidence suggests that the gut microbiome contributes to the pathophysiology of such inflammatory disorders [69,70,71,72]. In particular, dysbiosis is associated with the pathogenesis of food allergies (Table 1) [73,74,75,76,77,78,79,80,81,82,83,84].

Bacteroidetes and Firmicutes makeup 90% of the microbiota and are involved in the pathogenesis of FA. The association with FA has been identified especially for *Clostridia* species, able to increase the production of Treg, with resulting inhibition of allergic inflammation and promotion of oral tolerance. *Bacteroides fragilis*, a species of Bacteroidetes, was also capable of producing polysaccharide A (PSA), which increased the suppressive capacity of Treg cells and the production of IL-10 by Foxp3 + T cells in a murine study. Several studies identify a lower abundance of bacterial class Clostridia, phylum Firmicutes, in children with food allergy compared to healthy children [3]. 

Fazlollahi M. et al. studied 141 children with egg allergy compared with healthy controls, highlighting a preponderance of Lachnospiraceae, Streptococcaceae, and Leuconostocaceae in the early gut microbiome of children with egg allergy. The association found between the presence of the families of *Lachnospiraceae* and *Ruminococcaceae* and egg sensitization has, therefore, led the authors to identify the early diversity of the microbiota in egg-sensitized children as a target for preventive or therapeutic interventions [81].

**Figure 3:**
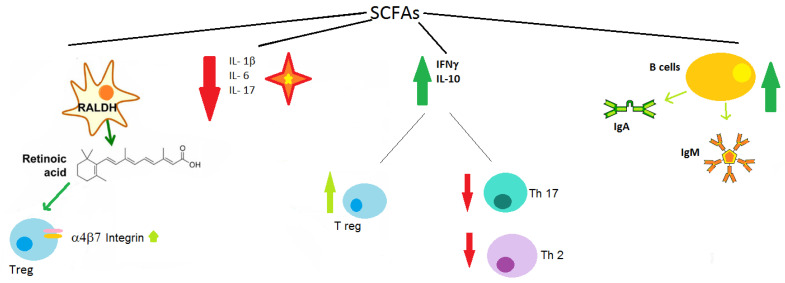
SCFAs effects in immunological tolerance: induce gut dendritic cells (DC) to express retinal aldehyde dehydrogenase (RALDH), with result in the production of retinoic acid that upregulates expression of the gut-homing integrins α4β7 on peripheral regulatory T cells (Treg); promote immune tolerance, regulate the antibody response through the production of IgA and IgM; stimulate the production of anti-inflammatory mediators such as IFNγ and IL-10, which induce the expansion of Treg cells and the suppression of proinflammatory T helper 17 (Th17) and Th2 cells; reduce the production of proinflammatory cytokines including IL-1β, IL-6, and IL-17.

Intestinal microorganisms such as *Clostridium leptum*, *Eubacterium rectal*, and *Faecalibacterium prausnitzii*, are able to produce SCFA, whose fermentation produces butyrate, propionate, acetate, and valerate with a higher concentration in the colon. SCFAs have direct immune-modulatory effects and are a key factor in promoting immunological tolerance towards harmless antigens and preventing inflammation (Figure 3).

In addition to the production of SCFA, intestinal bacteria also produce polyamines (PA) (spermidine, spermine, putrescine, cadaverine) essential for maintaining the intestinal barrier function through upregulation of junctional proteins. 

The rise of food allergy in modern society has led to the postulation of the hygiene hypothesis, according to which lack of early childhood exposure to infectious agents suppresses the development of the immune system with the rise of atopic diseases. Recent work has revisited the hygiene hypothesis model to include mode of delivery, antibiotic intake, diet, and synthetic chemicals as factors in altering gut microbiota [3]. After the introduction of the complementary diet, the composition of the microbiota varies according to the diet applied with evident compositional differences between a diet rich in fiber, characterized by *Alistipes*, *Bilophila*, and *Bacteroides*, and higher abundance of *Roseburia*, *Ruminococcus*, and *Eubacterium*, and diet rich in fat. In general, *Lactobacillus*, *Clostridium*, *Ruminococcus*, *Peptostreptococcus*, and *Bacteroides* are species that, through the catabolization of tryptophan, are able to regulate the immune response and the proliferation of T lymphocytes with consequent induction of the expression of the IL-10 receptor-1 (IL-10R1) and inhibition of proinflammatory cytokines [85]. In this context, it is clear that a diet that is higher in fat but low in fiber, like the Western one, maybe the cause of the increased prevalence of FA in Western countries [86].

About that, Mckenzie et al. have described the “nutrition-gut microbiome-physiology axis”, an essential link between diet, gut microbiota, and allergic diseases. It was also shown that food diversity was associated with greater expression of Foxp3, suggesting a protective effect of a diversified diet against the development of FA. On the contrary, reduced Foxp3 expression was present in children with a less diversified diet [1].

Data supporting the ability of the microbiota to influence allergic sensitization was found in mice treated with antibiotics and GF, which developed greater allergic sensitization than controls. In particular, Jiménez-Saiz R et al. demonstrated that eosinophil-deficient GF mice had intestinal fibrosis and were less prone to allergic sensitization than GF controls, establishing the role of the microbiota in regulating the frequency and activation of eosinophils in the intestinal mucosa [87].

More commonly, Clostridiales and Lactobacillales appear to have beneficial effects on food tolerance, while Bacteroidales and Enterobacteriales have ambivalent effects. In particular, children with AD and FA had a microbiota more colonized by *Escherichia coli* and *Bifidobacterium pseudocatenulatum* and less by *Bifidobacterium adolescentis*, *Akkermansia muciniphila*, *Bifidobacterium breve*, and *Faecalibacterium prausnitzii* compared to children with AD without FA. The authors, therefore, established an association, also in this case, between early colonization with potentially more pathogenic bacteria, such as *Clostridium difficile* or *Stafilococcus aureus*, and the development of FA, and *vice-versa* colonization with more beneficial bacteria such as *Bifidobacteria* and food tolerance [1]. In particular, studies in the literature have revealed the desensitizing effects of *Lactobacillus rhamnosus GG* in cow’s milk and in peanut allergy [3,88,89].

The World Allergy Organization (WAO), regarding probiotic supplementation, concluded that there was weak evidence to support their action in reducing the risk of developing allergic disorders in pediatric patients but that nevertheless, a small reduction in risk could be connected [90].

## 5. Discussion

The alteration of the microbiota appears to be a common ground for both OP and FA. In particular, an altered intestinal barrier can be considered common damage to both diseases. The intestinal barrier is defined as a functional unit that constantly balances the antigenic charge of the intestinal lumen with a complex immunological and non-immunological organization of the intestinal mucosa. It performs two fundamental functions for the survival of the individual: allow the absorption of nutrients and defend the body from the penetration of harmful macromolecules mediated by the tight junctions of the apical epithelial cells. It has recently been observed that tight junctions are regulated in their functioning by cytokines produced in the intestine and can be altered by various factors, including alcohol consumption, dietary imbalances, and the action of bacterial toxins [91]. Alteration of the intestinal barrier and the gut microbiota cause the development of an important inflammatory substrate in the intestine, which leads to FA and the loss of estrogen typical of primary OP. A study by Li et al. found that depletion of hormones increases inflammation in the intestine through a greater antigenic load that crosses the intestinal barrier [22]. Estrogens seem to have an ambivalent role in promoting the development of allergic diseases and the degranulation of mast cells in association with exposure to allergens. Andrè et al. also found involvement of the use of oral contraceptives in the etiology of urticaria and chronic angioneurotic edema [91].

The intestinal microbiota is also able to influence the estrogens circulating level through the secretion of β-glucuronidase, an enzyme that activates them. The integrity of the intestinal barrier, normally preserved by the presence of four phyla, Bacteriodetes, Firmicutes, Actinobacteria, and Proteobacteria, is altered by dysbiosis, in which the reduction in cell-cell junctions increases intestinal permeability, resulting in bacterial translocation inducing a systemic inflammatory state at the basis of various pathological processes. Furthermore, it is important to underline that dysbiosis leads to a reduced deconjugation of estrogens with a reduction in their circulation, leading to the activation of CD4 + T cells. CD4 + T cells produce RANKL, OPG, and TNF-α, promoting osteoclast activation and bone absorption through the OPG- RANK-RANKL signal transduction pathway.

There is evidence that the inflammatory process is at the basis of both OP and FA. Several proinflammatory and anti-inflammatory mediators are involved in the immunopathogenesis of FA, in which allergens can stimulate Th1, Th2, and Th17 cytokines in a heterogeneous way. Kara et al. [92] have hypothesized the monitoring of cytokines such as TNF-α and IL-6 in the follow-up of patients with FA, while Nadelkopoulou et al. [93] have investigated the role of IL-10 in the treatment of FA. TNF-a and IL-1 are also the main cytokines involved in bone metabolism and bone loss related to estrogen deficiency. The role of IL-33 is being debated [94], its activity contributing to the development of various allergological conditions through its action on mast cells, eosinophils, Th2 cells, Tregs, natural killers, basophils, dendritic cells, and activated macrophages, but at the same time appears to have a protective role on bone by inhibiting RANKL-dependent osteoclastogenesis [91,92,93,94,95,96,97,98,99].

Bone health is highly dependent on diet and nutritional style that determines the type of microbiota in host organisms. The intestinal microbiome, in fact, contributes to the production of proteins and enzymes related to digestion and energy metabolism since it ferments undigested nutrients transforming them into SCFA, leading to a decrease in intestinal permeability and greater absorption of minerals such as calcium. There are several factors linked to the microbiota that unite OP and FA; in particular, a key role is played by SCAF with immunomodulatory and anti-inflammatory effects, exercised through the promotion of immune tolerance, the production of IgA and IgM, the reduction in the production of proinflammatory cytokines including IL-1β, IL-6, and IL-17 [41], and the production of anti-inflammatory IFNγ and IL-10 with consequent expansion of Treg cells and suppression of proinflammatory Th17 and Th2 cells.

The strong influence of the diet on the microbiota and consequently on the OP and allergic manifestations has led to evaluate the fundamental role of nutrition. In fact, a diet rich in fat can reduce the absorption of essential elements for bone health, including vitamin D, K, C, and folate. Moreover, the high-fat diet of Western countries is one of the factors that can contribute to the increased prevalence of allergies in Western countries [100].

Diet-induced obesity has been demonstrated to be a factor of increased susceptibility for FA, and in particular, the microbiota associated with the high-fat diet was found to be able to increase the propensity for FA as evidence of the connection between diet-microbiota and FA [101]. It has been reported that heat-killed lactic acid bacteria (LAB) increased the percentage of peripheral CD4 + CD25 + Foxp3 + Treg cells and relieve symptoms in the pollen season when administered to patients with mild Japanese cedar pollinosis. Although not through the microbiota, LAB is thought to act directly on the immune system. In particular, increased Treg, along with SCFA, is considered a promising target for improving both allergies and bone metabolic balance [102]. Roduit et al. analyzed the levels of SCFA in fecal samples of 301 children at the age of 1, reporting that children with the highest levels of butyrate and propionate were less likely to suffer from asthma by the age of 3 and 6 years and showed significantly lower allergic sensitization with a decrease in food allergy risks and allergic rhinitis diagnosis. More recently, Cait et al. examined the role of bacterial butyrate production in the gut during early childhood in the development of atopic disease and concluded that the lack of genes encoding key enzymes for both the breakdown of carbohydrates and the production of butyrate was the basis of allergic sensitization [1]. 

Although OP and allergies are two conditions with a high prevalence in the general population and the relationship between fracture risk and allergic diseases such as asthma, atopic dermatitis, urticaria, FA is now well established, to date, we do not yet have adequate epidemiological studies on the prevalence of allergies in patients with OP [103,104,105,106,107,108,109]. This probably partially depends on the recent recognition of their interrelationship other than the difficulty in including all together so many “allergic” pathologies with such different peculiarities. The currently available data in the literature refer to single pathologies and limited series. Furthermore, the available studies evaluate the presence of OP in allergic diseases and not the prevalence of allergies in patients with OP. Even in recent studies conducted to evaluate the prevalence of comorbidities in subjects with OP, no data relating to allergies emerge [110], probably because there is still not enough awareness of the influence of allergic disease on bone health. Furthermore, the retrospective analysis conducted in Italy on 64.852 subjects at high risk for fracture collected between 2016 and 2020, through the DeFRAcalc79, does not take into account allergic diseases among the variables for calculating the risk of OP [111].

Vitamin D plays an important role in the pathogenesis of OP and also in the regulation of intestinal tight junctions, leading to the hypothesis that its deficiency may compromise the integrity of the barrier or induce alterations in the composition of the intestinal microbiota, increasing the risk of FA and of OP. Vitamin D, in fact, is important in the maintenance of bone health through the regulation of serum calcium homeostasis. The lack of vitamin D increases bone resorption in order to maintain the right serum calcium levels, making up for the lack of calcium reabsorbed by the gut induced by vitamin D deficiency [112].

Sardecka-Milewska et al. found that children with cow’s milk allergy have lower serum concentrations of vitamin D than healthy children [113]. The role of vitamin D in the development of FA is further confirmed by Koplin et al., who documented an attenuated association between low serum levels of vitamin D and food allergy only in subjects with polymorphisms associated with lower levels of vitamin D-binding protein. This involvement of vitamin D in FA could be linked to the ability of vitamin D to induce the expression of IL-10 by Treg cells, leading to oral tolerance and its maintenance [114,115]. 

Finally, it is also essential to remember the relationships between microbiota and microRNA (miRNA). The latter are small non-coding RNAs capable of regulating gene expression. The importance of the role of miRNAs in many pathological conditions [116,117,118], including allergies [119] and OP [120,121], is emerging. Being able to fully understand the relationship between miRNA and microbiota could allow to have new disease markers and pave the way for new targeted and personalized therapeutic strategies [122].

The importance of the unaltered microbiota is underlined by the fact that the growing tendency to use antibiotics leads to impaired intestinal absorption with a deficiency of minerals important for bone health, thus contributing to the development of OP, on the other hand, the use of antibiotics compromises the development of oral tolerance mechanisms leading to increased development of FA.

On this basis is founded the use of prebiotics and probiotics for beneficial modulation of the intestinal microbiota both in OP and in allergological pathologies. For example, it has been shown that LGG is able to reduce the expression of TNFα, IL-17, and RANKL in cells isolated from the small intestine and bone marrow of mice, decreasing bone resorption, and also have desensitizing effects in cow’s milk and peanut allergy [45,88]. The data in this review based on the current literature highlight how the microbiota and some bacterial species can influence the propensity to develop diseases, including allergies and OP. In particular, the supplementation of beneficial bacteria and diet corrections seems to improve the outcome and prevent the onset of these diseases. 

## 6. Conclusions

Although further studies are needed on the topic, current evidence shows the driving role of the microbiota and its modulation on both bone remodeling and allergic sensitization processes. The full understanding of the existing interplay between microbiota, bone metabolism, and allergies can design new pathophysiological scenarios and open new and stimulating horizons for preventive and therapeutic strategies.

## Figures and Tables

**Table 1:** Association between the most frequent food allergy and microbiota.

Food Allergy	Associated Bacteria
Cow’s milk	↓Clostridia, Firmicutes
Cow’s milk, egg, peanut	↑Enterobacteriaceae ↓Bacteroidaceae
Peanut	↓Clostridiales ↑Bacteroidales
Egg white, cow’s milk, wheat, peanut, soy bean	↓Bacteroidetes ↑Firmicutes

## Data Availability

Not applicable.
